# Pediatrics

**Published:** 1902-10

**Authors:** Maud J. Frye

**Affiliations:** Buffalo, N. Y.


### PROGRESS IN MEDICAL SCIENCE.

### Pediatrics.

CONDUCTED BY MAUD J. FRYE, M. D., Buffalo, N. Y.

### FEEDING OF PREMATURE INFANTS.

CHAPIN (*Theory and Practice of Infant Feeding*) says: The author has tried all kinds of incubators and believes that only those having a fresh air inlet connected with the outer air are safe to use. If such an incubator is not obtainable, an ordinary soap box may be improvised, in which the baby is placed done up in cotton and surrounded by hot water bottles. The top of this box may be partially covered with a sheet or towel, so arranged as to allow free access of air. The babies at first seem to do best at an average temperature of 85° to 90°. The less the infant is disturbed the better; at the same time proper cleanliness of the baby and incubator must be insisted on, as these infants are very vulnerable to infection of all kinds. The most important factor in raising them is to secure breast milk. The milk must be drawn from the breast after the colostrum period, the preferable time being from about eight days to a month or so postpartum. Usually this must be diluted one-half with boiled water or sugar water solution. The best way to feed is by means of a medicine dropper, drop by drop, very slowly, care being taken to see that one drop is swallowed before another is given. This extra care is required from the tendency of fluids to get in the windpipe. The author has seen a number of deaths from this cause, as shown by autopsy.

When the infant is too feeble to swallow, a catheter, used as a stomach tube must be employed. It can be passed without removing the baby from the incubator. As soon as the baby grows older and is strong enough, a small nipple may be substituted. The exact amount to be given a premature baby must depend upon the period of uterogestation and its apparent development. In one case, weighing only 2½ lbs., a dram every hour was given by the author with good results. In better favored cases, from 4 to 8 drams can be given every hour or two. If the infant thrives, in two or three weeks it can be given pure breast milk at two-hour intervals. Without breast milk the chances of a premature baby are very poor. Both the fat and proteids of cow's milk are digested with difficulty in these cases, so they must be given in very small amounts. The milk must be diluted to represent fat, 1 per cent. sugar, 3 per cent. proteids, one-third of 1 per cent. (one-tenth nine-ounce top milk, plus one-thirtieth sugar) of which a dram may be given every hour, to be gradually increased if tolerated. At the eighth month a little stronger mixture may be borne, such as fat, 1.5; sugar, 5, proteids, ½ to ¾ per cent. (one-eighth to one-fifth nine-ounce top milk and one-twenty-fifth sugar.) Feed from 4 to 6 drams every hour and a half. When ordinary cow's milk cannot be digested, a trial may be made of whey, expressed beef juice, egg water or highly diluted condensed milk.

### THE MANAGEMENT OF RHEUMATIC CHILDREN.

CRANDALL (*Archives of Pediatrics*, August, 1902,) presents a most excellent paper calling attention, first, to the frequent distribution of rheumatic symptoms in childhood through months or even years, and to symptoms which are rarely seen in adult life. He says: The management of the rheumatic child may be considered under four headings: clothing, exercise, diet, medication. The rheumatic child should wear flannel at all seasons, though in summer it may be of thin texture. Its value as a means of prevention is too well-established to be doubted. Cold and wet feet should be especially avoided. The exercise and out-door life of rheumatic children should receive particular attention. There is a strong tendency on the part of the mothers to confine such children to the house too closely and thus render them the more susceptible to cold. Days of damp, east winds, particularly if the ground be covered by slush or melting snow, are especially favorable for the development of rheumatism. Concerning diet the trend of opinion seems to be in the direction of restricting sugars and starches, and not so much the nitrogenous foods. The rheumatic child is prone to be anemic and a plain generous nourishing diet which contains some nitrogenous matter is best. For acute cases the writer allows milk, broths or plain soups, together with weak lemonade and oatmeal water for thirst, returning to a more general diet as soon as possible.

Proper care of the throat and the removal of adenoid growths and enlarged tonsils are important prophylactic measures. The writer uses the salicylic treatment for acute rheumatism and in children, with a marked rheumatic tendency, he believes that he wards off acute attacks by giving salicylate of soda for one or two weeks out of a month, continued for a long period of time. Children suffering from any rheumatic manifestation, even chorea, if the latter be severe, should be kept in bed to prevent possible heart complications. Give iron or iron and cod-liver oil early in convalescence.

---

### SUMMER DIARRHEA OF INFANTS.

SHUTT (*Jour. Am. Med. Assn.*, August 2, 1902,) discusses the cause, prevention and treatment of summer diarrhea. The treatment may be summarised as follows: clear out the alimentary canal. If there is no nausea, give castor oil; if there is, calomel in 1-10 gr. doses. Irrigate the large intestine, using the high enema with either a weak boric acid or normal salt solution. The temperature of the irrigation may be determined by the child's temperature. If the child vomits repeatedly the stomach may be washed out. The child, whether breast or bottle-fed, must be taken off milk at once, and not returned to it till the stools become normal. For the first twelve hours or more give no food, but allow cold boiled water to drink. Later, allow egg water, rice water, animal broths, barley water, either plain or dextrinised, and liquid peptonoids. The latter may be given in amounts of ½ to 1 teaspoonful in water every two hours till the stools become normal. For fever use cold sponging. After the bowels are thoroughly cleaned out bismuth subnitrate, in 10 gr. doses, every two hours, till the stools become dark, is usually given, and later, if the cause for diarrhea is dependent on increased peristalsis, small doses of opium in the form of Dover's powder or paregoric.

---

### ACUTE ARTICULAR RHEUMATISM IN AN INFANT.

Barcus (*Archives of Pediatrics*, January, 1902,) reports a case of acute articular rheumatism seen in an infant twenty-seven days old. There was no history of chorea or tonsillitis in the parents and no pus focus in the infant which could have caused an arthritis. When five months pregnant the mother had suffered from muscular rheumatism. The case presented the symptoms which go to make up the classic picture of rheumatism—the character of and successive joint affection, the sour sweat, the characteristic rheumatic temperature, the control of the symptoms by the salicylates, and probable heart complication.

